# The association between TSH and thyroid hormones in the normal or subclinical dysfunction range with left ventricular diastolic dysfunction

**DOI:** 10.1038/s41598-024-66096-9

**Published:** 2024-07-02

**Authors:** Ji Eun Jun, Tae Hyuk Kim, Sun Wook Kim, Jae Hoon Chung, Jae Hyeon Kim, You-Bin Lee, Mira Kang

**Affiliations:** 1 Department of Endocrinology and Metabolism, Kyung Hee University Hospital at Gangdong, Kyung Hee University College of Medicine, Seoul, Republic of Korea; 2 Division of Endocrinology and Metabolism, Department of Medicine, Thyroid Center, Samsung Medical Center, Sungkyunkwan University School of Medicine, 81 Irwon-Ro, Gangnam-Gu, Seoul, 06351 Republic of Korea; 3 Department of Health Promotion, Samsung Medical Center, Sungkyunkwan University School of Medicine, Seoul, Republic of Korea

**Keywords:** Thyroid hormone, Subclinical hypothyroidism, Subclinical thyrotoxicosis, Diastolic disfunction, Endocrine system and metabolic diseases, Thyroid diseases, Cardiology

## Abstract

Thyroid hormones modulate the cardiovascular system. However, the effects of subclinical thyroid dysfunction and euthyroidism on cardiac function remain unclear. We investigated the association between left ventricular (LV) diastolic dysfunction and subclinical thyroid dysfunction or thyroid hormones within the reference range. This cross-sectional study included 26,289 participants (22,197 euthyroid, 3,671 with subclinical hypothyroidism, and 421 with subclinical thyrotoxicosis) who underwent regular health check-ups in the Republic of Korea. Individuals with thyroid stimulating hormone (TSH) levels > 4.2 µIU/mL and normal free thyroxine (FT4, 0.78–1.85 ng/dL) and triiodothyronine (T3, 76–190 ng/dL) levels were defined as having subclinical hypothyroidism. Individuals with serum TSH levels < 0.4 µIU/mL and normal FT4 and T3 levels were defined as having subclinical thyrotoxicosis. The cardiac structure and function were evaluated using echocardiography. LV diastolic dysfunction with normal ejection fraction (EF) was defined as follows: EF of > 50% and (a) E/e’ ratio > 15, or (b) E/e’ ratio of 8–15 and left atrial volume index ≥ 34 mL/m^2^. Subclinical hypothyroidism was significantly associated with cardiac indices regarding LV diastolic dysfunction. The odds of having LV diastolic dysfunction was also increased in participants with subclinical hypothyroidism (adjusted odds ratio [AOR] 1.36, 95% confidence interval [CI], 1.01–1.89) compared to euthyroid participants. Subclinical thyrotoxicosis was not associated with LV diastolic dysfunction. Among the thyroid hormones, only serum T3 was significantly and inversely associated with LV diastolic dysfunction even within the normal range. Subclinical hypothyroidism was significantly associated with LV diastolic dysfunction, whereas subclinical thyrotoxicosis was not. Serum T3 is a relatively important contributor to LV diastolic dysfunction compared to TSH or FT4.

## Introduction

Thyroid hormones modulate physiological functions of the cardiovascular system in various ways. Thyroid hormones, particularly the biologically active triiodothyronine (T3), affect myocardial contractility, heart rate, and systemic vascular resistance^1^, and thus both thyroid hormone excess and deficiency can be associated with a higher cardiovascular risk^1,2^. However, few clinical studies have evaluated the association between thyroid hormone levels and left ventricular (LV) diastolic dysfunction.

Studies suggest a potential association between primary thyroid dysfunction and LV diastolic dysfunction, with a prevalence as high as 35%^3^. However, the aforementioned study, may have overestimated the prevalence of hypothyroidism due to two factors: a higher proportion of elderly participants and a narrow reference range for thyroid-stimulating hormone (TSH). Compared with overt thyroid dysfunction, subclinical dysfunction or changes within the normal range still lack sufficient evidence for their association with cardiovascular disease (CVD)^4,5^. However, a growing body of observational data suggests that cardiovascular risk may also be increased in patients with subclinical thyroid dysfunction^6,7^.

Given the central role of thyroid hormones in the cardiovascular system, it is important to elucidate their association with LV diastolic dysfunction to explore their potential as therapeutic targets. Therefore, this study aimed to investigate the association between thyroid function in addition to each hormone level and LV diastolic dysfunction in a large cohort with euthyroid status or subclinical thyroid dysfunction.

## Methods

### Study design and population

This study employs a retrospective cross-sectional design to investigate the association between thyroid hormone levels and LV diastolic dysfunction. A total of 40,125 participants aged ≥ 20 years who underwent comprehensive health examinations including echocardiography, between September 2006 and December 2013 at the Samsung Medical Centre's Health Promotion Centre in Seoul, Republic of Korea were included. We excluded 4,082 participants with missing or erroneous echocardiography data, 3,782 participants with incomplete thyroid function test (TFT) results (TSH, total T3, and free thyroxine [FT4]), and 4,621 participants with missing clinical data other than TFT. Participants with an estimated glomerular filtration rate of < 30 mL/min/1.73 m^2^ (N = 51) or a left ventricular ejection fraction (LVEF) of < 50% (N = 113) were also excluded. Among them, 512 participants were excluded for the following reasons: overt hypothyroidism (N = 118), overt thyrotoxicosis (N = 206), atypical TFT, such as a non-thyroidal illness pattern with an isolated increase or decrease in T3/FT4 (N = 188), and medications for any thyroid disease (N = 675). Finally, 26,289 participants (22,197 with euthyroidism, 3,671 with subclinical hypothyroidism, and 421 with subclinical thyrotoxicosis) were enrolled (Supplementary Fig. 1). None of the enrolled individuals was diagnosed with chronic diseases such as cancer or inflammatory, renal, hepatic, cardiac, thyroid, or neurological diseases.

This study was approved by the Institutional Review Board (IRB no. 2023-03-008) of Samsung Medical Center. The requirement for informed consent was waived by the Institutional Review Board of Samsung Medical Center due to the retrospective nature of the study. All methods were carried out in accordance with relevant institutional guidelines and regulations.

### Definitions

Euthyroid status was defined as TSH within the reference range of 0.4–4.2 µIU/mL using an immunoradiometric assay kit (Immunotech, Marseille Cedex, France), T3 within the reference range of 76–190 ng/dL, and FT4 within the reference range of 0.78–1.85 ng/dL using a commercialized radioimmunoassay kit (Immunotech)^8^. Participants with serum TSH levels above the upper limit of the reference range (> 4.2 µIU/mL) with normal FT4 and T3 levels were considered to have subclinical hypothyroidism. Subclinical hypothyroidism was further categorized based on the degree of TSH elevation, as either mild (TSH > 4.2 and < 10 µIU/mL) or severe (TSH ≥ 10 µIU/mL)^9^. Participants with TSH levels below the lower limit of the reference range (< 0.4 µIU/mL) with normal FT4 and T3 levels were considered to have subclinical thyrotoxicosis, which was further categorized as either mild (TSH > 0.1 and < 0.4 µIU/mL) or severe (TSH ≤ 0.1 µIU/mL)^4^. Blood samples for TSH, FT4, and T3 concentrations were collected predominantly from 9 to 11 am after an overnight fast to minimize diurnal variation.

Diabetes mellitus (DM) was defined as a fasting plasma glucose (FPG) concentration of 126 mg/dL or higher, an HbA1c level of 6.5% or higher, or the use of any antidiabetic medication according to the American Diabetes Association criteria^10^. Hypertension was defined as a systolic blood pressure (BP) of 140 mmHg or greater, diastolic BP of 90 mmHg or greater, or the use of any antihypertensive medication^11^. Dyslipidemia was diagnosed in individuals who satisfied one of the following criteria: low-density lipoprotein cholesterol level, ≥ 160 mg/dL; high-density lipoprotein cholesterol level, < 40 mg/dL; or triglyceride level, ≥ 200 mg/dL. Individuals taking any medication for dyslipidemia were also classified as having dyslipidemia^12^.

### Echocardiographic data and definition of diastolic dysfunction

Echocardiography was performed by trained sonographers and clinicians using Vivid 7 (GE Medical Systems, Milwaukee, WI, USA) following standardized guidelines. Echocardiographic parameters including EF, left atrial volume index (LAVI, reflecting LA enlargement), transmitral early diastolic velocity (E), and mitral annular early diastolic velocity (e′) were simultaneously assessed. LVEF was assessed using the biplane Simpson rule via manual tracing of digital images^13^.

Linear measurements of the left posterior wall thickness (PWT), intraventricular septal thickness (IVST), and LV cavity diameters at end diastole (LVIDd) and systole (LVIDs) were obtained in the parasternal long-axis M-mode. LV mass was calculated according to the following equation: LV mass (g) = 0.8 × [1.04 × (LVIDd + IVST + PWT)^3^ − LVIDs^3^] + 0.6^14^. LV mass index (LVMI) was calculated as LV mass divided by body surface area. Relative wall thickness (RWT) was derived as 2 × PWT/LVID, and a high RWT was considered as a ratio > 0.42 in line with the American Society of Echocardiography (ASE) recommendations^15^.

Pulse-wave Doppler was used to measure mitral inflow in the apical five-chamber view, which included the peak transmitral inflow velocity during early diastole (E) and late diastole (A), E/A ratio, and deceleration time (DT) of early diastolic flow. The mitral E/e ratio was used as an index of the LV diastolic filling pressure. LV diastolic dysfunction with normal EF was defined as follows: EF of > 50% and (a) E/e’ ratio greater than 15 or (b) E/e’ ratio of 8–15 and LAVI greater than or equal to 34 mL/m^2^ ^16–18^.

LV diastolic dysfunction was further classified into four grades (0: normal, 1: mild, 2: moderate, 3: severe) according to two definitions; a simplified definition based on E/e′ only^19^ and the ASE 2009^17^.

### Statistical analysis

Mean ± standard deviation and median (interquartile range [IQR]: 25th-75th percentile) are used to describe normally and non-normally distributed data, respectively. Percentages are used for categorical variables. Analysis of variance for continuous variables and the χ^2^ test for categorical variables were used to compare baseline parameters and echogenic indices according to thyroid function. Post hoc comparisons with the euthyroid group for each group were performed using Bonferroni’s correction.

We used multiple linear regression analyses to assess the relationship between thyroid hormones and indices of cardiac structure, as well as between thyroid hormones and cardiogenic function. Multiple logistic regression analysis was performed to evaluate the odds ratios (ORs) and 95% confidence intervals (CIs), according to thyroid hormone concentration or thyroid function status for the risk of LV diastolic dysfunction. Confounding factors used for the analyses were selected from clinically important variables with a *P*-value < 0.1 in univariate analyses, and a maximum Variance Inflation Factor of 5 was used to exclude multicollinearity problems. Finally, we constructed two adjusted models: a model adjusted for age and sex (model 1), and a model further adjusted for body mass index (BMI), hypertension, diabetes, dyslipidemia, and current smoking (model 2). Structural coefficients were calculated, and a relative weights analysis with significance tests was performed to determine the relative importance of each independent variable in the multivariate model^20,21^. Stepwise selection assigned a weight that reflected the contribution of each variable to the outcome, and the relative importance is expressed as a percentage.

All *P*-values are two-tailed, with < 0.05 considered significant. Statistical Package for the Social Sciences version 26.0 for Windows (IBM Corp., Armonk, NY, USA) was used for all statistical analyses.

## Results

### Baseline characteristics according to thyroid functions

Among the 26,289 participants, 22,197 (84.4%) had normal thyroid function, 3,671 (14.0%) had subclinical hypothyroidism, and 421 (1.6%) had subclinical thyrotoxicosis. Baseline clinical and biochemical characteristics of the study population, according to thyroid function, are summarized in Table 1. Participants with subclinical hypothyroidism or thyrotoxicosis were generally older, less obese, had lower proportions of men and current smokers, lower diastolic BP, and were less likely to use hypertension, dyslipidemia, or diabetes medications than those with normal thyroid function. FPG levels were slightly lower in the subclinical hypothyroidism group; however, HbA1c levels and prevalence of diabetes did not differ from those in the euthyroid group. The prevalence of diabetes and dyslipidemia were lower in the subclinical thyrotoxicosis group than in the euthyroid group.

**Table 1:** Clinical characteristics of the study participants.

Variables	Total (N = 26,289)	Normal thyroid (N = 22,197)	SC-hypothyroid (N = 3,671)	SC-thyrotoxicosis (N = 421)	*P* value (ANOVA)
Age, years	55.7 ± 9.7	55.4 ± 9.7	57.0 ± 10.0***	57.0 ± 9.9**	< 0.001
Male, *N* (%)	18,600 (70.8)	16,200 (73.0)	2166 (59.0)***	234 (55.6)***	< 0.001
BMI, kg/m^2^	24.3 ± 2.9	24.4 ± 2.9	24.1 ± 2.9***	23.9 ± 2.8*	< 0.001
Waist circumference, cm	Male: 87.9 ± 7.7	87.9 ± 7.7	87.9 ± 8.1	87.1 ± 7.1	0.268
	Female: 80.4 ± 8.2	80.4 ± 8.2	80.4 ± 8.4	80.2 ± 7.9	0.921
Fasting plasma glucose, mg/dL	98.7 ± 19.7	98.8 ± 19.9	97.8 ± 18.4**	98.5 ± 19.8	0.011
HbA1c, %	5.72 ± 0.76	5.72 ± 0.76	5.70 ± 0.74	5.68 ± 0.66	0.207
Total cholesterol, mg/dL	197.0 ± 35.1	196.9 ± 35.0	198.4 ± 35.4*	190.8 ± 36.0**	< 0.001
Triglycerides, mg/dL	130.8 ± 77.7	131.1 ± 77.9	130.0 ± 76.4	126.6 ± 72.9	0.402
HDL-C, mg/dL	53.76 ± 14.1	53.5 ± 14.1	54.2 ± 14.2*	54.7 ± 16.0	0.007
LDL-C, mg/dL	124.1 ± 31.0	124.0 ± 30.9	125.3 ± 31.3*	117.3 ± 31.6***	< 0.001
HS C-reactive protein, mg/dL	0.06 (0.03–0.11)	0.06 (0.03–0.12)	0.05 (0.03–0.11)	0.06 (0.03–0.11)	0.106
Estimated GFR, ml/min/1.73m^2^	83.3 ± 14.3	83.7 ± 14.2	80.5 ± 14.5***	87.5 ± 14.5***	< 0.001
Diabetes, N (%)	3444 (13.1)	2946 (13.3)	453 (12.3)	45 (10.7)*	0.032
Dyslipidemia, N (%)	10,106 (38.4)	8580 (38.7)	1380 (37.6)	146 (34.7)*	0.045
Current smoking, N (%)	6354 (24.2)	5471 (24.6)	797 (21.7)***	86 (20.4)***	< 0.001
Systolic BP, mmHg	121.9 ± 16.9	121.8 ± 16.9	122.1 ± 17.0	122.7 ± 16.3	0.449
Diastolic BP, mmHg	76.1 ± 10.8	76.3 ± 10.8	75.0 ± 11.0***	74.4 ± 10.5**	< 0.001
Hypertension, N (%)	10,032 (38.2)	8504 (38.3)	1373 (37.4)	155 (36.8)	0.254
Medication of hypertension, yes, N (%)	6854 (26.1)	5795 (26.1)	952 (25.9)*	107 (25.4)*	0.001
Medication of dyslipidemia, yes, N (%)	2162 (8.2)	1856 (8.4)	274 (7.5)**	32 (7.6)*	0.001
Medication of diabetes, yes, N (%)	2149 (8.2)	1833 (8.3)	291 (7.9)**	25 (5.9)*	0.001
Thyroid function tests					
TSH, µIU/ml	2.22 (1.43–3.30)	1.99 (1.37–2.77)	5.36 (4.64–6.70)***	0.18 (0.06–0.32)***	< 0.001
FT4, ng/dL	1.26 ± 0.19	1.26 ± 0.18	1.19 ± 0.18***	1.39 ± 0.25***	< 0.001
T3, ng/dL	115.5 ± 18.9	115.3 ± 18.7	116.1 ± 19.7	119.7 ± 23.1***	< 0.001

Data was presented as mean ± standard deviation, interquartile range, or number (percentage).

*BMI* body mass index, *HDL-C* high-density lipoprotein cholesterol, *LDL-C* low-density lipoprotein cholesterol, *HS* high-sensitivity, *TSH* thyroid-stimulating hormone, *FT4* free thyroxine, *T3* triiodothyronine, *SC* subclinical, *ANOVA* analysis of variance.

*P < 0.05, **P < 0.01, and ***P < 0.001 for comparing the euthyroid group.

### Thyroid functions and echogenic indices

Participants with subclinical hypothyroidism showed significantly higher LVPWd (12.7 ± 8.4 vs. 12.3 ± 8.1 mm, *P* = 0.009) and larger proportions of high RWT (28.1 vs. 25.6%, *P* = 0.005) than those with normal thyroid function (Table 2). Mitral E/e’ velocity ratio was significantly higher in subclinical hypothyroidism group (8.1 ± 2.7 vs. 7.9 ± 2.4, *P* < 0.001), while other indices of diastolic function, such as E/A ratio, or DT did not differ between the two groups (Table 2). In addition to mitral E/e’, the mitral E/A ratio was found to be significantly increased in subjects with severe subclinical hypothyroidism (1.86 ± 1.49 vs. 0.98 ± 1.95, *P* < 0.001), as defined by TSH levels of ≥ 10 µIU/mL (Supplementary Table 1).

**Table 2:** Echogenic indices according to thyroid function.

	Euthyroid (N = 22,197)	SC-hypothyroidism (N = 3671)	SC-thyrotoxicosis (N = 421)
LAVI, mL/m^2^	27.2 ± 7.8	27.2 ± 8.2	27.1 ± 8.3
LV geometry			
LVMI, g/m^2^	84.3 ± 17.0	83.1 ± 17.2	84.0 ± 16.5
LVIDd, mm	48.6 ± 4.1	48.8 ± 4.2	48.7 ± 4.3
LVPWd, mm	12.3 ± 8.1	12.7 ± 8.4**	13.2 ± 9.1*
RWT, cm	0.38 (0.33–0.43)	0.38 (0.33–0.44)	0.37 (0.33–0.45)
> 0.42, N (%)	5672 (25.6)	1031 (28.1)**	121 (28.7)**
IVSd, cm	8.9 ± 1.2	8.8 ± 1.3	8.8 ± 1.1
Systolic function			
Ejection fraction, %	65.9 ± 5.5	66.1 ± 5.6	66.3 ± 5.3
Diastolic function			
Mitral E/A ratio	0.98 ± 1.95	1.03 ± 4.15	0.92 ± 0.27
Mitral E/e’	7.9 ± 2.4	8.1 ± 2.7***	8.3 ± 2.3**
> 15, N (%)	212 (0.9)	50 (1.4)*	5 (1.2)
DT, ms	224.7 ± 47.3	224.8 ± 47.2	221.7 ± 48.2

*LAVI* left atrial volume index, *LVMI* left ventricular mass index, *LVIDd* left ventricular internal diameter at diastole, *LVPWd* left ventricular posterior wall thickness at end-diastole, *RWT* relative wall thickness, *IVSd* diastolic interventricular septum, *DT* deceleration time.

*P < 0.05, **P < 0.01, and ***P < 0.001 for comparing the euthyroid group.

The subclinical thyrotoxicosis group had significantly higher LVPWd (13.2 ± 9.1 vs. 12.3 ± 8.1 mm, *P* = 0.032) and a larger proportion of high RWT (28.7 vs. 25.6%, *P* = 0.008). Only the mitral E/e’ velocity ratio (8.3 ± 2.3 vs. 7.9 ± 2.4, *P* = 0.005) was significantly increased among the indices of LV diastolic dysfunction compared to the euthyroid participants (Table 2). The severe subclinical thyrotoxicosis group showed a markedly increased E/e′ (8.5 ± 2.4 vs. 7.9 ± 2.4, *P* = 0.003) and a significantly decreased DT (213.2 ± 45.1 vs. 224.7 ± 47.3 ms, *P* = 0.003), whereas the mild subclinical thyrotoxicosis group did not differ in echogenic indices and LV diastolic dysfunction markers (Supplementary Table 2).

### Thyroid hormone concentrations and echogenic indices

Linear associations between TSH, FT4, and T3 levels and echogenic indices of the cardiac structure are shown in Table 3. TSH levels were inversely associated with LVMI in the adjusted models, while they were positively associated with LAVI, LVIDd, LVPWd and RWT. Serum FT4 levels were inversely associated with LVMI, LVIDd, and RWT, while they were positively associated with LAVI in the adjusted models, while they were not associated with LVPWd and IVSd. Serum T3 levels were inversely associated with LAVI, LVMI, and LVIDd in both the unadjusted and adjusted models, but were not associated with LVPWd, RWT, and IVSd.

**Table 3:** Linear association between thyroid hormone levels and cardiac structure indices.

	TSH		FT4		T3	
	Standardized* β*	*P* value	Standardized* β*	*P* value	Standardized* β*	*P* value
LAVI						
Unadjusted	0.001	0.999	− 0.032	< 0.001	− 0.045	< 0.001
Model 1	0.016	0.007	0.011	0.077	− 0.033	< 0.001
Model 2	0.015	0.016	0.012	0.057	− 0.041	< 0.001
LVMI						
Unadjusted	− 0.033	< 0.001	− 0.056	< 0.001	− 0.054	< 0.001
Model 1	− 0.021	0.003	− 0.044	< 0.001	− 0.046	< 0.001
Model 2	− 0.021	0.003	− 0.044	< 0.001	− 0.058	< 0.001
LVIDd						
Unadjusted	0.044	< 0.001	− 0.003	0.597	− 0.049	< 0.001
Model 1	0.012	0.040	− 0.049	< 0.001	− 0.054	< 0.001
Model 2	0.014	0.016	− 0.041	< 0.001	− 0.067	< 0.001
LVPWd						
Unadjusted	0.016	0.012	− 0.008	0.214	− 0.006	0.314
Model 1	0.023	< 0.001	− 0.017	0.008	− 0.007	0.280
Model 2	0.015	0.015	− 0.011	0.077	− 0.005	0.393
RWT						
Unadjusted	0.022	< 0.001	− 0.015	0.015	− 0.007	0.225
Model 1	0.025	< 0.001	− 0.019	0.002	− 0.008	0.211
Model 2	0.017	0.006	− 0.014	0.023	− 0.004	0.513
IVSd						
Unadjusted	− 0.036	< 0.001	0.020	0.002	0.008	0.173
Model 1	− 0.009	0.140	− 0.006	0.351	0.011	0.066
Model 2	− 0.009	0.099	− 0.002	0.759	− 0.008	0.149

Model 1 was adjusted for age and sex.

Model 2 was adjusted for age, sex, body mass index, hypertension, diabetes, dyslipidemia, and current smoking status.

*LAVI* left atrial volume index, *LVMI* left ventricular mass index, *LVIDd* left ventricular internal diameter at diastole, *LVPWd* left ventricular posterior wall thickness at end-diastole, *RWT* relative wall thickness, *IVSd* diastolic interventricular septum, *TSH* thyroid-stimulating hormone, *FT4* free thyroxine, *T3* triiodothyronine.

Linear associations between TSH, FT4, and T3 levels and echogenic indices of cardiac function are shown in Table 4. Among the indices of LV diastolic dysfunction, the TSH level was positively associated with the E/A ratio in the adjusted models. The FT4 level was inversely associated with the E/A ratio, and E/E’ velocity ratio in the adjusted models, whereas it was positively associated with DT. The serum T3 level was inversely associated with the E/A ratio and E/E’ velocity ratio in the adjusted models, while it was positively associated with DT.

**Table 4:** Linear association between thyroid hormone levels and indices of left ventricular diastolic function.

	TSH		FT4		T3	
	Standardized β	p value	Standardized β	p value	Standardized β	*P* value
Mitral E/A ratio						
Unadjusted	0.011	0.073	− 0.003	0.605	− 0.021	0.001
Model 1	0.013	0.032	− 0.014	0.026	− 0.024	< 0.001
Model 2	0.013	0.040	− 0.014	0.025	− 0.023	< 0.001
Mitral E/e’						
Unadjusted	0.016	0.010	− 0.087	< 0.001	− 0.036	< 0.001
Model 1	− 0.008	0.185	− 0.021	0.001	− 0.015	0.008
Model 2	− 0.009	0.098	− 0.021	< 0.001	− 0.030	< 0.001
DT						
Unadjusted	− 0.002	0.710	0.001	0.813	0.011	0.085
Model 1	− 0.004	0.557	0.031	< 0.001	0.023	< 0.001
Model 2	− 0.002	0.688	0.030	< 0.001	0.022	< 0.001

Model 1 was adjusted for age and sex. Model 2 was adjusted for age, sex, body mass index, hypertension, diabetes, dyslipidemia, and current smoking status.

*TSH* thyroid-stimulating hormone, *FT4* free thyroxine, *T3* triiodothyronine, *DT* deceleration time.

### Association between thyroid function/thyroid hormone concentration and the presence of LV diastolic dysfunction

**Figure 1:**
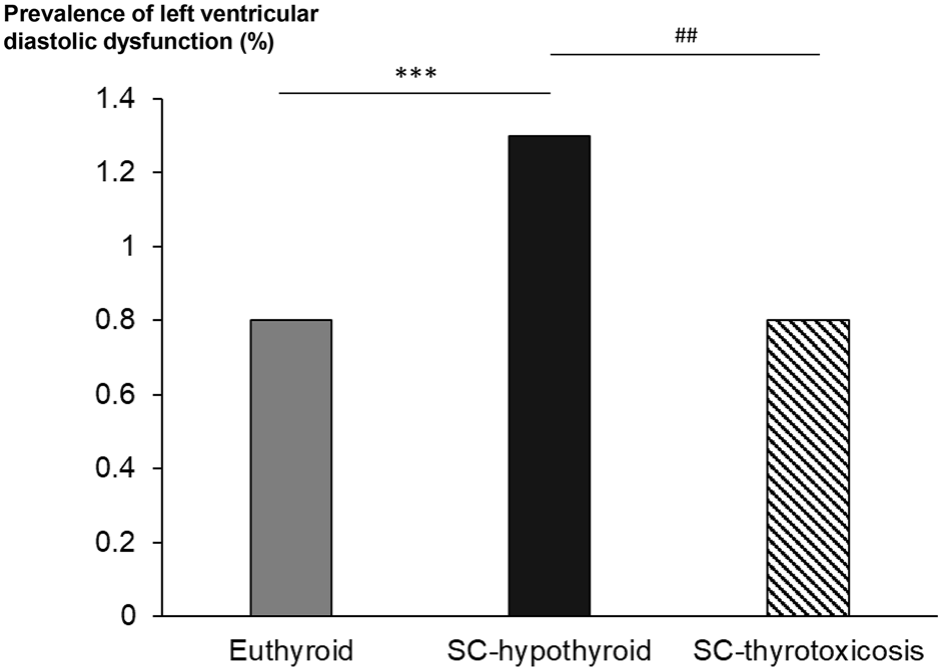
Prevalence of LV diastolic dysfunction among euthyroid, subclinical hypothyroid, and subclinical thyrotoxicosis status. SC, subclinical.

The prevalence of LV diastolic dysfunction was significantly higher in the subclinical hypothyroid group than in the euthyroid group (1.3% vs. 0.8%, *P* < 0.001) or subclinical thyrotoxicosis group (1.3% vs. 0.8%, *P* = 0.013) (Fig. 1).

In a multivariate logistic regression model (Table 5, model 2), TSH per 1.0 µIU/mL increase (OR, 1.05; 95% CI, 1.01–1.09, *P* = 0.022) and T3 per 10.0 ng/dL decrease (OR, 1.14; 95% CI, 1.05–1.23, *P* = 0.001) were significantly associated with increased risk of LV diastolic dysfunction. However, the FT4 levels were not associated with LV diastolic dysfunction. Compared to the euthyroid participants, those with subclinical hypothyroidism had a significantly higher risk of LV diastolic dysfunction (OR, 1.36; 95% CI, 1.01–1.89, *P* = 0.049) and the risk was even greater when the TSH level was 10.0 µIU/mL and more (OR, 1.99; 95% CI, 1.07–5.00, *P* = 0.047), while those with subclinical thyrotoxicosis were not associated with LV diastolic dysfunction. Among the euthyroid participants, only T3 level (OR, 1.14; 95% CI, 1.04–1.24, *P* = 0.006) was significantly and inversely associated with the risk of LV diastolic dysfunction in a multivariate model (Table 5, model 2), while TSH and FT4 levels were not. Low T3 level was significantly correlated with the presence of LV diastolic dysfunction in both sexes; however, it was significant only in male individuals among the euthyroid population.

**Table 5:** Odds ratios for the presence of left ventricular diastolic dysfunction according to thyroid hormone levels and function.

	Unadjusted		Model 1		Model 2	
	**OR (95% CI)**	**P value**	**OR (95% CI)**	**P value**	**OR (95% CI)**	**P value**
Total subjects (N = 26,289)						
TSH (per 1 µIU/ml increase)	1.05 (1.02–1.09)	0.001	1.05 (1.01–1.09)	0.025	1.05 (1.01–1.09)	0.022
FT4 (per 1 ng/dL decrease)	2.11 (1.01–4.44)	0.048	1.62 (0.77–3.38)	0.202	1.56 (0.74–3.29)	0.242
T3 (per 10 ng/dL decrease)	1.16 (1.07–1.25)	< 0.001	1.11 (1.03–1.20)	0.005	1.14 (1.05–1.23)	0.001
SC-hypothyroid (ref. euthyroid)						
TSH > 4.2 µIU/ml	1.81 (1.31–2.51)	< 0.001	1.36 (1.01–1.89)	0.049	1.36 (1.01–1.89)	0.049
TSH ≥ 10.0 µIU/ml	2.48 (1.01–6.08)	0.048	1.90 (1.07–4.74)	0.049	1.99 (1.07–5.00)	0.047
SC-thyrotoxicosis (ref. euthyroid)						
TSH < 0.4 µIU/ml	1.31 (0.48–3.55)	0.594	0.91 (0.33–2.51)	0.861	0.93 (0.34–2.56)	0.884
TSH ≤ 0.1 µIU/ml	2.77 (0.87–8.78)	0.083	2.32 (0.71–7.55)	0.164	2.49 (0.76–8.19)	0.134
Euthyroid subjects (N = 22,197)						
TSH (per 1 µIU/ml increase)	1.02 (0.86–1.21)	0.809	0.97 (0.82–1.15)	0.726	0.99 (0.83–1.17)	0.865
FT4 (per 1 ng/dL decrease)	1.77 (0.74–4.22)	0.199	0.52 (0.22–1.25)	0.143	0.56 (0.23–1.36)	0.201
T3 (per 10 ng/dL decrease)	1.16 (1.06–1.26)	0.001	1.11 (1.02–1.21)	0.019	1.14 (1.04–1.24)	0.006

Model 1 was adjusted for age and sex. Model 2 was adjusted for age, sex, body mass index,  hypertension, diabetes, dyslipidemia, and current smoking status.

*TSH* thyroid-stimulating hormone, *FT4* free thyroxine, *T3* triiodothyronine, *DT* deceleration time, *OR* odds ratio, *CI* confidence interval, *SC* subclinical.

Serum T3 level was also significantly and inversely correlated with the severity of LV diastolic dysfunction in a multivariate regression model regardless of different definitions (standardized *β* = -0.020, *P* = 0.001 by simplified definition, and standardized *β* = -0.012, *P* = 0.045 by ASE 2009 definition), whereas TSH and FT4 levels were not (Supplementary Table 3).

**Figure 2:**
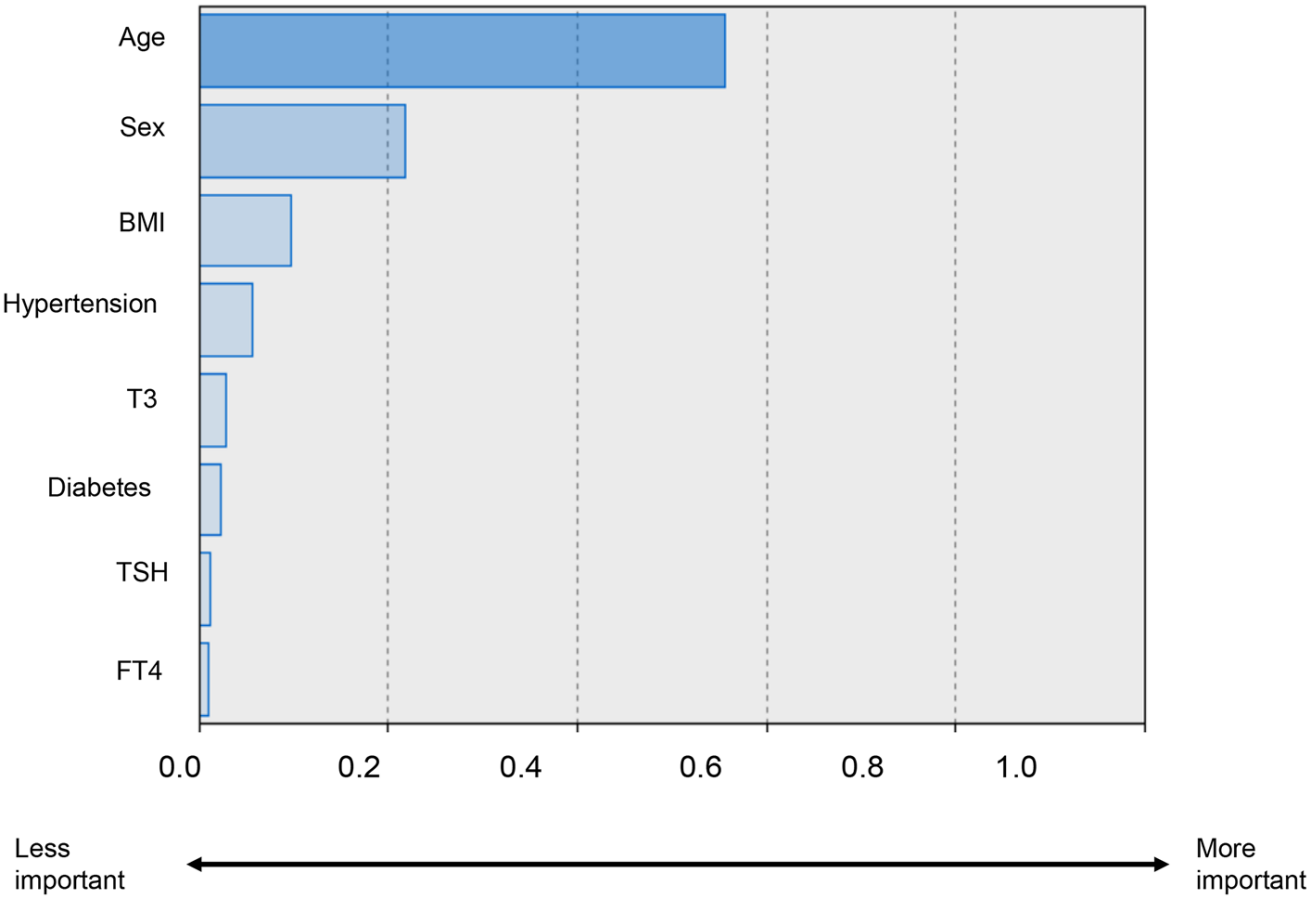
Contribution to the presence of left ventricular diastolic dysfunction among various risk factors including thyroid hormones. The closer to value of 1.0, the more important factor of left ventricular diastolic dysfunction. BMI, body mass index; T3, triiodothyronine; TSH, thyroid-stimulating hormone.

Among the variables included in the multivariate logistic regression models, age (56.7% of contribution) was the most significant contributing factor for LV diastolic dysfunction (Fig. 2). The serum T3 level (3.4% of contribution) was a more important factor for LV diastolic dysfunction than the TSH (1.4% of contribution) or FT4 levels (1.1% of contribution), although its relative importance was modest (Fig. 2).

## Discussion

In the present study, we found that subclinical hypothyroidism was significantly associated with the presence of LV diastolic dysfunction, while subclinical thyrotoxicosis was not associated with the presence of LV diastolic dysfunction. Thyroid hormone levels within the normal reference range and subclinical status were inversely associated with the indices of cardiac structure and diastolic function, whereas TSH levels were positively associated. Differences in the echogenic indices were more pronounced between severe subclinical thyroid dysfunction group and euthyroid subjects compared to the total subclinical group versus euthyroid subjects.

The effects of thyroid hormones on cardiac function are mediated by genomic and non-genomic mechanisms^22^. Thyroid hormones upregulate the expression of genes encoding sodium/potassium-transporting ATPases, increase the transcription of the myosin heavy chain (MHC) α gene, resulting in an increased contraction velocity^23,24^. In particular, T3 upregulates the cardiac sarcoplasmic/endoplasmic reticulum calcium ATPase 2 (*SERCA2*) gene via nuclear receptors^25^, whereas low T3 levels induce the downregulation of *SERCA2*, which correlates with impaired calcium reuptake. This is thought to contribute to the development of diastolic dysfunction in the failing myocardium^26,27^. The non-genomic effects of thyroid hormones activate sodium, potassium, and calcium membrane channels, which are involved in the signaling pathways of cardiomyocytes and vascular smooth muscle cells^1,28^. Taken together, thyroid hormones, particularly T3, increase cardiac output through positive inotropic and chronotropic effects and decrease systemic vascular resistance through vasodilation^22^.

T3, a biologically active form of thyroid hormone, plays a central role in regulating cardiac function. In this study, subtle changes in the serum T3 levels within the normal reference range, but not in the FT4 or TSH levels, were inversely and significantly associated with the presence of LV diastolic dysfunction. The influence of T3 on LV diastolic dysfunction was more prominent than that of FT4 or TSH, thereby supporting the aforementioned pathophysiology. Besides, as an extracardiac effect, T3 may modulate metabolic deterioration as an adaptive process^29^. Despite enrolling relatively healthy adults for a check-up, we cannot entirely rule out the possibility of non-thyroidal illness in some participants with LV diastolic dysfunction and low T3 levels.

Overt hypothyroidism decreases cardiac output, myocardial contractility, relaxation, and heart rate, while increasing systemic vascular resistance^30^. Subclinical hypothyroidism also partially presents the same cardiovascular changes as overt hypothyroidism^31,32^. Therefore, subclinical hypothyroidism is associated with LV diastolic dysfunction, mainly due to impaired ventricular filling and relaxation^33–36^, rather than morphological changes in the heart. A meta-analysis also suggested that cardiac systolic and diastolic function, but not cardiac structure, improved after levothyroxine supplementation^37^. However, individuals with subclinical hypothyroidism in this study exhibited elevated LVPWd and RWT despite no significant difference in LVMI, indicating concentric remodeling^38^. Another pilot study also reported that LVPWd was slightly yet significantly increased in a subclinical hypothyroid group (TSH, 4.2–9.9 µIU/mL) compared with a euthyroid group, and it significantly decreased after levothyroxine therapy^39^. In the Cardiovascular Health Study^40^, individuals with TSH levels ranging from 10.0–19.9 µIU/mL had greater LV mass and LV diastolic dysfunction indices compared to euthyroid individuals, whereas no differences were observed between those with TSH levels of 4.5–9.9 µIU/mL and those with normal TSH levels. These findings suggest that severe subclinical hypothyroidism may alter both cardiac structure and function. Our study observed significantly higher LVPWd and RWT as indices of cardiac structure and mitral E/e′ as an index of cardiac function due to the inclusion of a larger number of individuals with severe subclinical hypothyroidism (7.7% of total participants) and a more severe degree of subclinical hypothyroidism (TSH > 20 µIU/mL) than in the Cardiovascular Health Study. Mitral E/A ratio was also elevated solely in participants with TSH ≥ 10.0 µIU/mL compared to the euthyroid participants.

Thyrotoxicosis is associated with a hyperdynamic state characterized by tachycardia, increased cardiac preload and contractility, and diminished systemic vascular resistance^41^. Thus, it causes high cardiac output, LV hypertrophy, and enhanced diastolic function in the early stage, followed by biventricular dilatation and congestive heart failure in the later stages^42^. Nonetheless, inconsistent results regarding the association between overt/subclinical thyrotoxicosis and LV diastolic dysfunction have been reported. Some studies have found increased LV mass in patients with subclinical thyrotoxicosis^43–45^, while others have not^46,47^. Reduced e’ indicating LV diastolic dysfunction was exclusively present in hyperthyroid patients aged ≥ 40 years^47^. This study found an increase in LVPWd and RWT, as well as a greater E/e′, suggesting that subclinical thyrotoxicosis may have an effect on both cardiac structure and function, similar to subclinical hypothyroidism. Severe subclinical thyrotoxicosis (TSH ≤ 0.1 µIU/mL) additionally presented elevated mitral E/e′, indicating LV diastolic dysfunction. However, in contrast to subclinical hypothyroidism, subclinical thyrotoxicosis itself was not associated with the presence of LV diastolic dysfunction.

Current guidelines recommend starting thyroid hormone treatment for subclinical hypothyroidism if the TSH level is greater than 10 µIU/mL, and considering treatment in those with increased CVD risk when the serum TSH level is 4.5–10 µIU/mL, regardless of the patient’s age^9,48^. LV diastolic dysfunction is reversible with thyroid hormone replacement in subclinical hypothyroidism^33,37^ or levothyroxine dose reduction in exogenous subclinical thyrotoxicosis^45^. Compatible with the importance of T3 on LV diastolic dysfunction, T3 replacement at euthyroid levels improved both systolic and diastolic functions in an animal model^49^. This indicates that, even without primary thyroid disease or abnormal hormone levels, T3 supplementation may have beneficial effects^45^. In fact, a low free T3/FT4 ratio, which is a potential indicator of poor outcomes in patients with heart failure and preserved EF^50^, was found to normalize in more than 70% of patients with hypothyroidism after thyroidectomy with levothyroxine and liothyronine combination therapy. In contrast, levothyroxine alone did not normalize the free T3/FT4 ratio^51^.

This study has several limitations. First, the enrolled participants were recruited from a single hospital; thus, they do not represent the general Korean population. Additionally, patients with thyroid autoimmunity or transient abnormalities in thyroid function could not be excluded from the study population. Second, the cross-sectional design of this study hindered the establishment of causal relationships, and the data did not consider the changes in thyroid hormone levels that occur throughout the entire range of heart failure in individuals with normal thyroid function. In addition, transient and persistent thyroid dysfunction could not be distinguished. Third, data on the etiology, duration of subclinical thyroid dysfunction, and concomitant medications affecting thyroid function were lacking. Fourth, residual confounding factors, such as medications affecting thyroid and cardiac functions, cannot be thoroughly excluded. Fourth, the definition of diastolic dysfunction in this study was based on the guidelines used in 2009^17^ and 2016^16^, owing to the lack of echographic parameters for tricuspid regurgitation. Fifth, the presence of established clinical risk factors, including age, sex, BMI, and hypertension, is known to have a more substantial influence on LV diastolic dysfunction compared to the independent effect of T3, which is more likely to be modest. Lastly, brain natriuretic peptide (BNP), a reliable marker of heart failure, was not measured simultaneously, even though BNP levels can be affected by serum T3 levels^52^.

## Conclusion

Decreasing thyroid function (higher TSH, lower FT4, or lower T3 levels) is associated with LV diastolic dysfunction, and subclinical hypothyroidism is significantly associated with the presence of LV diastolic dysfunction. Subclinical thyrotoxicosis is, in part, associated with changes in the indices of LV structure or function, but fails to achieve statistical significance in association with the presence of LV diastolic dysfunction. Furthermore, we observed notable correlations between indices of LV diastolic dysfunction and severe subclinical thyroid dysfunction, rather than mild thyroid dysfunction. Although the influence of serum T3 on LV diastolic dysfunction cannot exceed that of conventional risk factors such as age, this study highlights a significant inverse association between subtle changes in T3 levels, even within the reference range, and LV diastolic dysfunction. This finding also aligns with the established role of other risk factors, such as overt thyroid dysfunction. Further preclinical and clinical studies should focus on investigating the fundamental biology related to thyroid dysfunction in the development and improvement of LV diastolic dysfunction through treatment with thyroid hormones or targeted thyromimetic analogs.

### Supplementary Information

Supplementary Tables.

Supplementary Figures.

## Data Availability

The datasets generated during and/or analyzed during the current study are not publicly available due to our institutional policy but are available from the corresponding author on reasonable request.

## References

[1] Klein I, Ojamaa K (2001). Thyroid hormone and the cardiovascular system. N. Engl. J. Med..

[2] Yamakawa H (2021). Thyroid hormone plays an important role in cardiac function: From bench to bedside. Front. Physiol..

[3] Favuzzi AMR (2020). Hormonal deficiencies in heart failure with preserved ejection fraction: Prevalence and impact on diastolic dysfunction: A pilot study. Eur. Rev. Med. Pharmacol. Sci..

[4] Surks MI (2004). Subclinical thyroid disease: Scientific review and guidelines for diagnosis and management. JAMA.

[5] Helfand, M. & U. S. Preventive Services Task Force. Screening for subclinical thyroid dysfunction in nonpregnant adults: a summary of the evidence for the U.S. Preventive Services Task Force. *Ann. Intern. Med.* **140**, 128–141, 10.7326/0003-4819-140-2-200401200-00015 (2004). 10.7326/0003-4819-140-2-200401200-00015 14734337

[6] Cappola AR (2019). Thyroid and cardiovascular disease research agenda for enhancing knowledge, prevention, and treatment. Circulation.

[7] Cooper DS, Biondi B (2012). Subclinical thyroid disease. Lancet.

[8] Jun JE (2017). Association between changes in thyroid hormones and incident type 2 diabetes: A seven-year longitudinal study. Thyroid.

[9] Pearce SH (2013). 2013 ETA guideline: Management of subclinical hypothyroidism. Eur Thyroid J.

[10] Elsayed NA (2022). Classification and diagnosis of diabetes: Standards of care in diabetes—2023. Diabetes Care.

[11] Messerli FH, Williams B, Ritz E (2007). Essential hypertension. Lancet.

[12] Rhee EJ (2020). Prevalence and current management of cardiovascular risk factors in korean adults based on fact sheets. Endocrinol. Metab. (Seoul).

[13] Russo C (2010). Comparison of echocardiographic single-plane versus biplane method in the assessment of left atrial volume and validation by real time three-dimensional echocardiography. J. Am. Soc. Echocardiogr..

[14] Devereux RB (1986). Echocardiographic assessment of left ventricular hypertrophy: Comparison to necropsy findings. Am. J. Cardiol..

[15] Lang RM (2015). Recommendations for cardiac chamber quantification by echocardiography in adults: an update from the American Society of Echocardiography and the European Association of Cardiovascular Imaging. J. Am. Soc. Echocardiogr..

[16] Nagueh SF (2016). Recommendations for the evaluation of left ventricular diastolic function by echocardiography: An update from the American Society of Echocardiography and the European Association of cardiovascular imaging. J. Am. Soc. Echocardiogr..

[17] Nagueh SF (2009). Recommendations for the evaluation of left ventricular diastolic function by echocardiography. J. Am. Soc. Echocardiogr..

[18] Yoo JH (2021). Relationship between low skeletal muscle mass, sarcopenic obesity and left ventricular diastolic dysfunction in Korean adults. Diabetes/Metabol. Res. Rev..

[19] Lanspa MJ (2019). A simplified definition of diastolic function in sepsis, compared against standard definitions. J. Intensive Care.

[20] Thomas DR, Zhu P, Zumbo BD, Dutta S (2008). On measuring the relative importance of explanatory variables in a logistic regression. J. Mod. Appl. Stat. Methods.

[21] Lorenzo-Seva U, Ferrando PJ (2011). FIRE: An SPSS program for variable selection in multiple linear regression analysis via the relative importance of predictors. Behav. Res. Methods.

[22] Vale C, Neves JS, von Hafe M, Borges-Canha M, Leite-Moreira A (2019). The role of thyroid hormones in heart failure. Cardiovasc. Drugs Ther..

[23] Kaasik A, Paju K, Vetter R, Seppet EK (1997). Thyroid hormones increase the contractility but suppress the effects of beta-adrenergic agonist by decreasing phospholamban expression in rat atria. Cardiovasc. Res..

[24] Holt E, Sjaastad I, Lunde PK, Christensen G, Sejersted OM (1999). Thyroid hormone control of contraction and the Ca^(2+)^-ATPase/phospholamban complex in adult rat ventricular myocytes. J. Mol. Cell. Cardiol..

[25] Rohrer D, Dillmann WH (1988). Thyroid hormone markedly increases the mRNA coding for sarcoplasmic reticulum Ca^2+^-ATPase in the rat heart. J. Biol. Chem..

[26] Kranias EG, Hajjar RJ (2012). Modulation of cardiac contractility by the phospholamban/SERCA2a regulatome. Circ. Res..

[27] Grais IM, Sowers JR (2014). Thyroid and the heart. Am. J. Med..

[28] Ojamaa K, Klemperer JD, Klein I (1996). Acute effects of thyroid hormone on vascular smooth muscle. Thyroid.

[29] Jun JE (2017). Hormetic effect of triiodothyronine in metabolically healthy obese persons. Endocrine.

[30] Jabbar A (2017). Thyroid hormones and cardiovascular disease. Nat. Rev. Cardiol..

[31] Neves JS (2020). Thyroid hormones and modulation of diastolic function: A promising target for heart failure with preserved ejection fraction. Ther. Adv. Endocrinol. Metab..

[32] Chen X, Zhang N, Cai Y, Shi J (2013). Evaluation of left ventricular diastolic function using tissue Doppler echocardiography and conventional doppler echocardiography in patients with subclinical hypothyroidism aged < 60 years: A meta-analysis. J. Cardiol..

[33] Biondi B (1999). Left ventricular diastolic dysfunction in patients with subclinical hypothyroidism. J. Clin. Endocrinol. Metabol..

[34] Kosar F (2005). Evaluation of right and left ventricular function using pulsed-wave tissue Doppler echocardiography in patients with subclinical hypothyroidism. J. Endocrinol. Invest..

[35] Turhan S (2006). Effects of thyroxine therapy on right ventricular systolic and diastolic function in patients with subclinical hypothyroidism: A study by pulsed wave tissue Doppler imaging. J. Clin. Endocrinol. Metabol..

[36] Vitale G (2002). Left ventricular myocardial impairment in subclinical hypothyroidism assessed by a new ultrasound tool: Pulsed tissue Doppler. J. Clin. Endocrinol. Metabol..

[37] Wang X (2022). Effect of levothyroxine supplementation on the cardiac morphology and function in patients with subclinical hypothyroidism: A systematic review and meta-analysis. J. Clin. Endocrinol. Metabol..

[38] Hashem MS (2015). Left ventricular relative wall thickness versus left ventricular mass index in non-cardioembolic stroke patients. Medicine (Baltimore).

[39] Erkan G (2011). The evaluation of diastolic dysfunction with tissue Doppler echocardiography in women with subclinical hypothyroidism and the effect of L-thyroxine treatment on diastolic dysfunction: A pilot study. J. Thyroid Res..

[40] Rodondi N (2008). Subclinical thyroid dysfunction, cardiac function, and the risk of heart failure. The Cardiovascular Health study. J. Am. College Cardiol..

[41] Khan R (2020). Thyroid and cardiovascular disease: A focused review on the impact of hyperthyroidism in heart failure. Cardiol. Res..

[42] Osuna PM, Udovcic M, Sharma MD (2017). Hyperthyroidism and the Heart. Methodist DeBakey Cardiovasc. J..

[43] Biondi B (2000). Endogenous subclinical hyperthyroidism affects quality of life and cardiac morphology and function in young and middle-aged patients. J. Clin. Endocrinol. Metabol..

[44] Shapiro LE (1997). Minimal cardiac effects in asymptomatic athyreotic patients chronically treated with thyrotropin-suppressive doses of L-thyroxine. J. Clin. Endocrinol. Metabol..

[45] Smit JW (2005). Reversible diastolic dysfunction after long-term exogenous subclinical hyperthyroidism: A randomized, placebo-controlled study. J. Clin. Endocrinol. Metabol..

[46] Iqbal A (2007). Thyroid stimulating hormone and left ventricular function. J. Clin. Endocrinol. Metabol..

[47] Yue WS (2011). Hyperthyroidism-induced left ventricular diastolic dysfunction: implication in hyperthyroidism-related heart failure. Clin. Endocrinol. (Oxf).

[48] Jonklaas J (2014). Guidelines for the treatment of hypothyroidism: Prepared by the american thyroid association task force on thyroid hormone replacement. Thyroid.

[49] Henderson KK (2009). Physiological replacement of T3 improves left ventricular function in an animal model of myocardial infarction-induced congestive heart failure. Circ. Heart Fail..

[50] Leite AR (2023). Clinical and pathophysiologic insights of free triiodothyronine/free thyroxine ratio in patients with heart failure with preserved ejection fraction: Data from the NETDiamond cohort. Cardiology.

[51] Brigante G (2024). Randomized double-blind placebo-controlled trial on levothyroxine and liothyronine combination therapy in totally thyroidectomized subjects: The LEVOLIO study. Eur. J. Endocrinol..

[52] Selvaraj S (2012). Association of serum triiodothyronine with B-type natriuretic peptide and severe left ventricular diastolic dysfunction in heart failure with preserved ejection fraction. Am. J. Cardiol..

