# Supplementary material for: The association between TSH and thyroid hormones in the normal or subclinical dysfunction range with left ventricular diastolic dysfunction
Source: Sci Rep. 2024 Jul 2;14:15169. doi: 10.1038/s41598-024-66096-9 (PMC11219717; doi:10.1038/s41598-024-66096-9)
Supplement: Supplementary file 1 — Supplementary Tables. [file 41598_2024_66096_MOESM1_ESM.docx]

**Table S1.** Echogenic indices between euthyroid and severe subclinical hypothyroidism

|  | Euthyroid  (*N* = 22197) | Severe SC-hypothyroidism  (TSH ≥ 10 uIU/ml)  (*N* = 282) | *P* value |
| --- | --- | --- | --- |
| *LAVI, mL/m^2^* | 27.2 ± 7.8 | 26.8 ± 7.9 | 0.393 |
| *LV geometry* |  |  |  |
| LVMI, g/m^2^ | 84.3 ± 17.0 | 82.0 ± 19.6 | 0.078 |
| LVIDd, mm | 48.6 ± 4.1 | 49.9 ± 4.6 | 0.003 |
| LVPWd, mm | 12.3 ± 8.1 | 13.3 ± 8.8 | 0.034 |
| RWT, cm | 0.38 (0.33 – 0.43) | 0.38 (0.33 – 0.47) | 0.122 |
| > 0.42, n (%) | 5672 (25.6) | 93 (33.6) | 0.005 |
| IVSd, cm | 8.9 ± 1.2 | 8.8 ± 1.4 | 0.100 |
| *Systolic function* |  |  |  |
| Ejection fraction, % | 65.9 ± 5.5 | 66.3 ± 5.8 | 0.260 |
| *Diastolic function* |  |  |  |
| Mitral E/A ratio | 0.98 ± 1.95 | 1.86 ± 1.49 | <0.001 |
| Mitral E/e’ | 7.9 ± 2.4 | 8.1 ± 3.2^*^ | 0.045 |
| >15, n (%) | 212 (0.9) | 5 (1.8) | 0.147 |
| DT, ms | 224.7 ± 47.3 | 225.5 ± 56.1 | 0.774 |

SC, subclinical; LAVI, left atrial volume index; LVMI, left ventricular mass index; LVIDd, left ventricular internal diameter of diastole; LVPwd, left ventricular posterior wall thickness at end-diastole; RWT, relative wall thickness; IVSd, diastolic interventricular septum; DT, deceleration time.

^*^*P* <0.05,^**^*P* <0.01, and ^***^*P* <0.001 for comparing euthyroid group.

**Table S2.** Echogenic indices between euthyroid and severe subclinical thyrotoxicosis

|  | Euthyroid  (*N* = 22197) | Severe SC-thyrotoxicosis  (TSH ≤ 0.10 uIU/ml)  (*N* = 151) | *P* value |
| --- | --- | --- | --- |
| *LAVI, mL/m^2^* | 27.2 ± 7.8 | 27.3 ± 9.3 | 0.816 |
| *LV geometry* |  |  |  |
| LVMI, g/m^2^ | 84.3 ± 17.0 | 85.3 ± 15.3 | 0.585 |
| LVIDd, mm | 48.6 ± 4.1 | 48.7 ± 4.0 | 0.926 |
| LVPWd, mm | 12.3 ± 8.1 | 13.6 ± 9.3 | 0.042 |
| RWT, cm | 0.38 (0.33 – 0.43) | 0.38 (0.33 – 0.47) | 0.885 |
| > 0.42, n (%) | 5672 (25.6) | 37 (24.5) | 0.696 |
| IVSd, cm | 8.9 ± 1.2 | 8.9 ± 1.2 | 0.500 |
| *Systolic function* |  |  |  |
| Ejection fraction, % | 65.9 ± 5.5 | 66.4 ± 5.0 | 0.237 |
| *Diastolic function* |  |  |  |
| Mitral E/A ratio | 0.98 ± 1.95 | 0.93 ± 0.26 | 0.735 |
| Mitral E/e’ | 7.9 ± 2.4 | 8.5 ± 2.4 | 0.003 |
| >15, n (%) | 212 (0.9) | 2 (1.3) | 0.618 |
| DT, ms | 224.7 ± 47.3 | 213.2 ± 45.1 | 0.003 |

SC, subclinical; LAVI, left atrial volume index; LVMI, left ventricular mass index; LVIDd, left ventricular internal diameter of diastole; LVPwd, left ventricular posterior wall thickness at end-diastole; RWT, relative wall thickness; IVSd, diastolic interventricular septum; DT, deceleration time.

^*^*P* <0.05, ^**^*P* <0.01, and ^***^*P* <0.001 for comparing euthyroid group.

|  | Univariate | | Multivariate^#^ | |
| --- | --- | --- | --- | --- |
|  | Standardized *β* | *P* value | Standardized *β* | *P* value |
| *Simplified definition* |  |  |  |  |
| TSH | 0.023 | <0.001 | 0.015 | 0.017 |
| FT4 | -0.015 | 0.012 | 0.008 | 0.206 |
| T3 | -0.023 | <0.001 | -0.020 | 0.001 |
| *ASE 2009 definition* |  |  |  |  |
| TSH | 0.015 | <0.001 | 0.009 | 0.143 |
| FT4 | -0.004 | 0.523 | 0.015 | 0.069 |
| T3 | -0.015 | 0.016 | -0.012 | 0.045 |
| ^#^Model was adjusted for age, sex, BMI, hypertension, diabetes, dyslipidemia, and current smoking status.  ASE, the American Society of Echocardiography. | | | | |

**Table S3.** Multiple regression analyses for the association between thyroid hormones and the severity of LV diastolic dysfunction
