# Supplementary figures and images for: The association between TSH and thyroid hormones in the normal or subclinical dysfunction range with left ventricular diastolic dysfunction
Source: Sci Rep. 2024 Jul 2;14:15169. doi: 10.1038/s41598-024-66096-9 (PMC11219717; doi:10.1038/s41598-024-66096-9)

## Slide 1
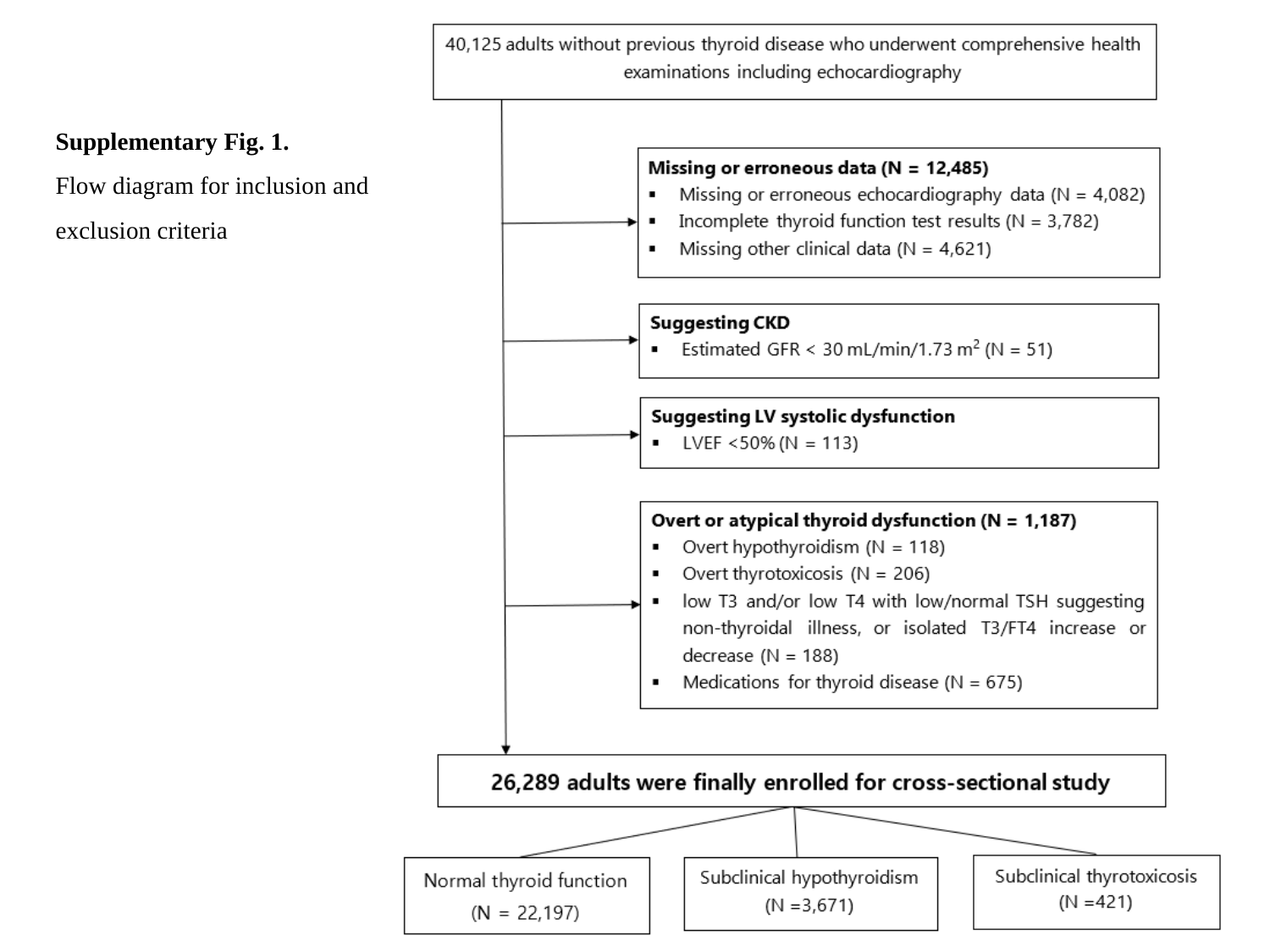

Supplementary Fig. 1.
Flow diagram for inclusion and exclusion criteria

Supplement: Supplementary file 2 — Supplementary Figures. [file 41598_2024_66096_MOESM2_ESM.pptx]
